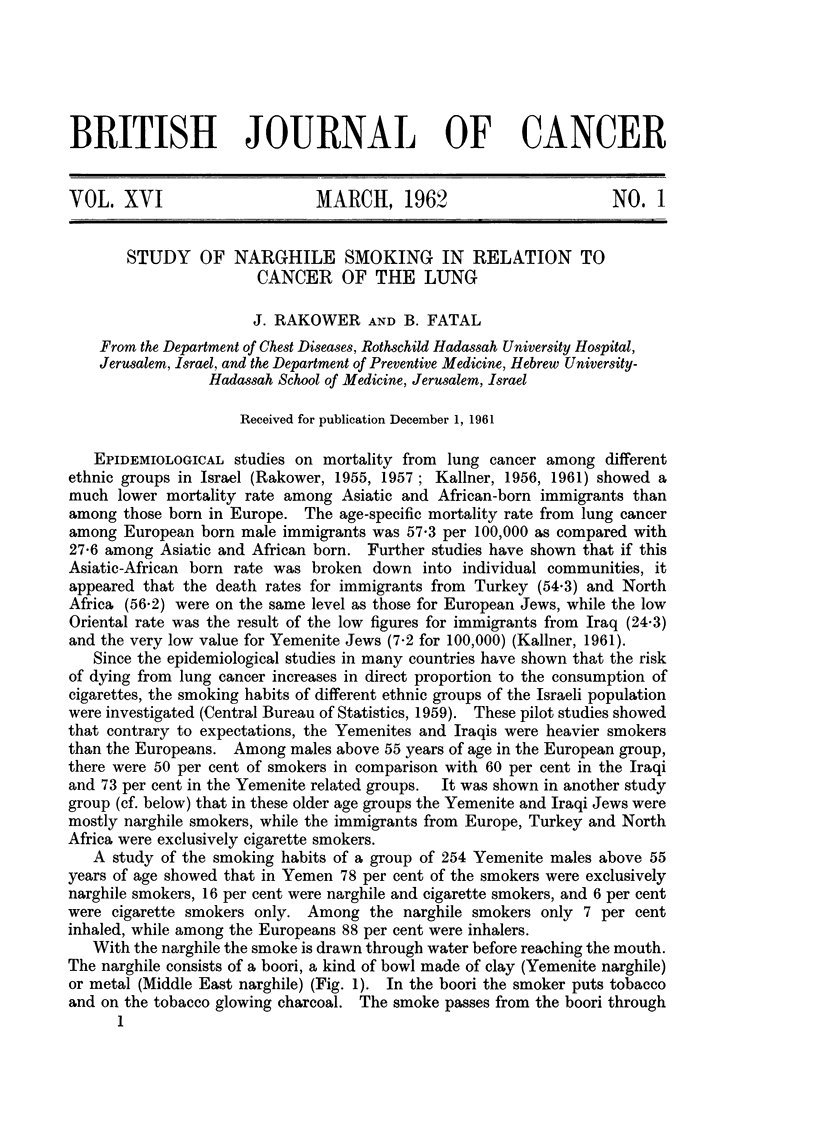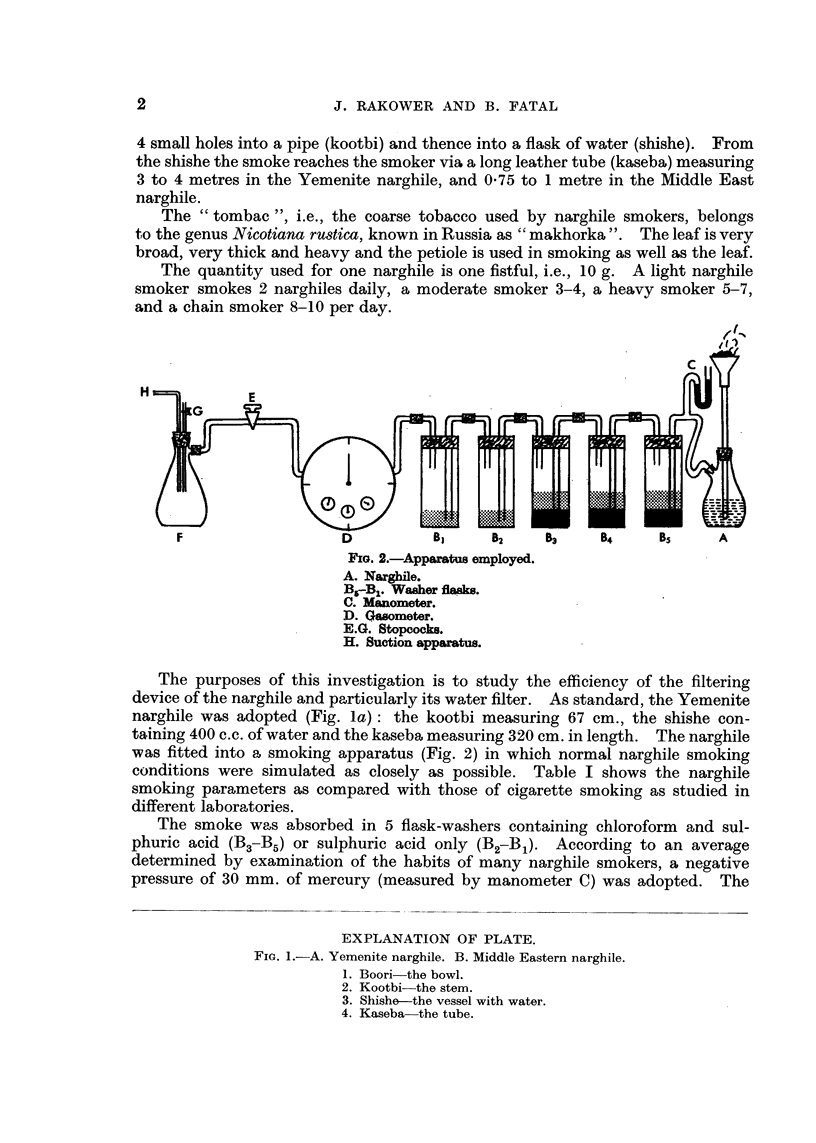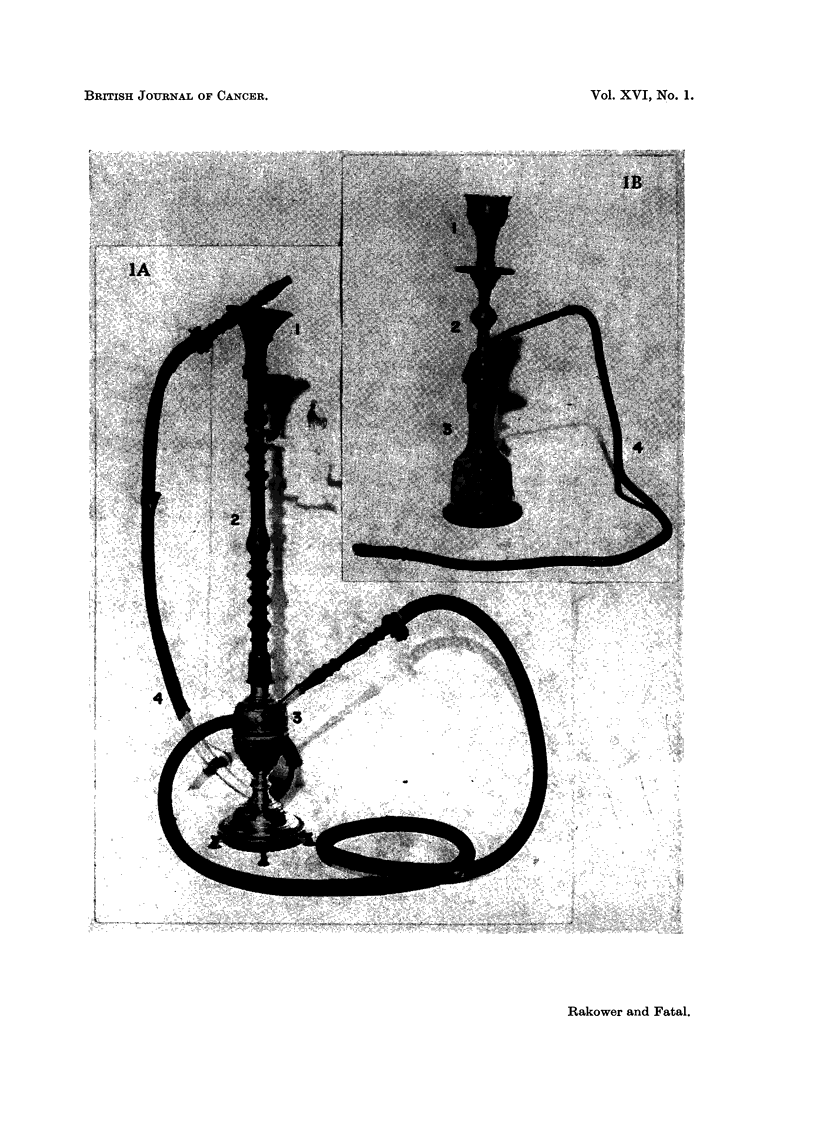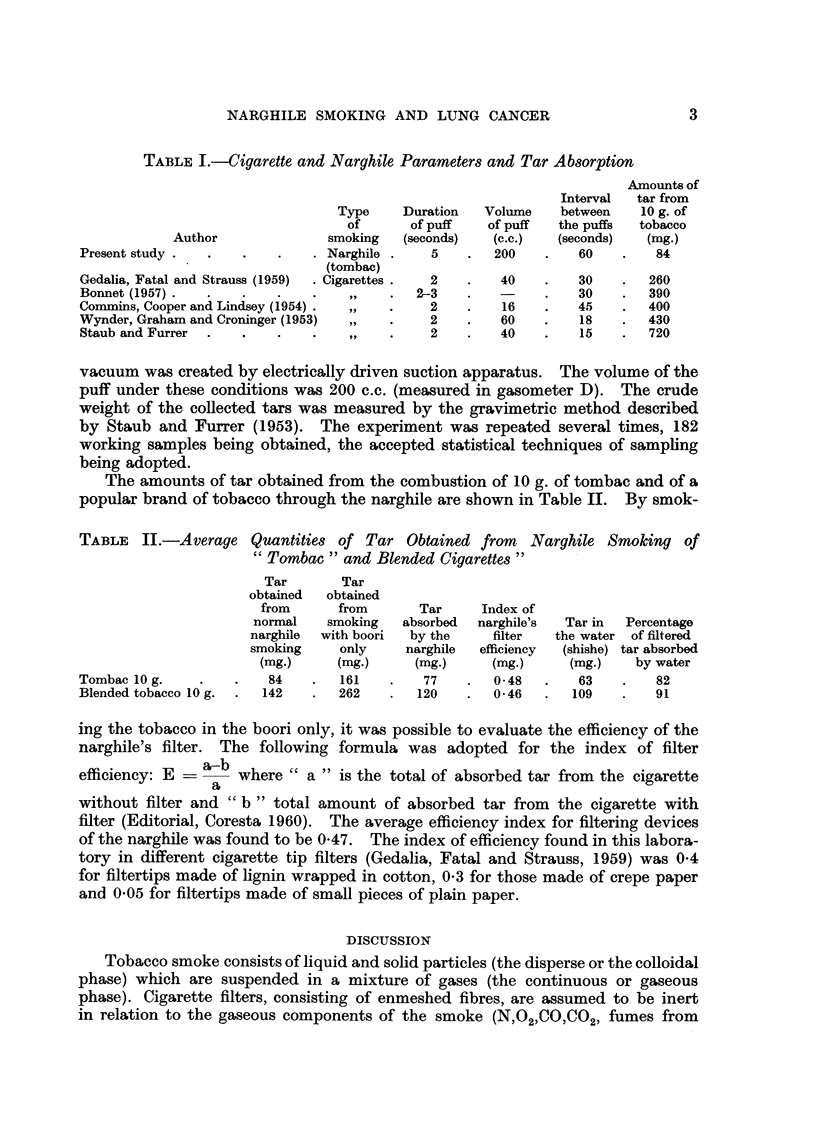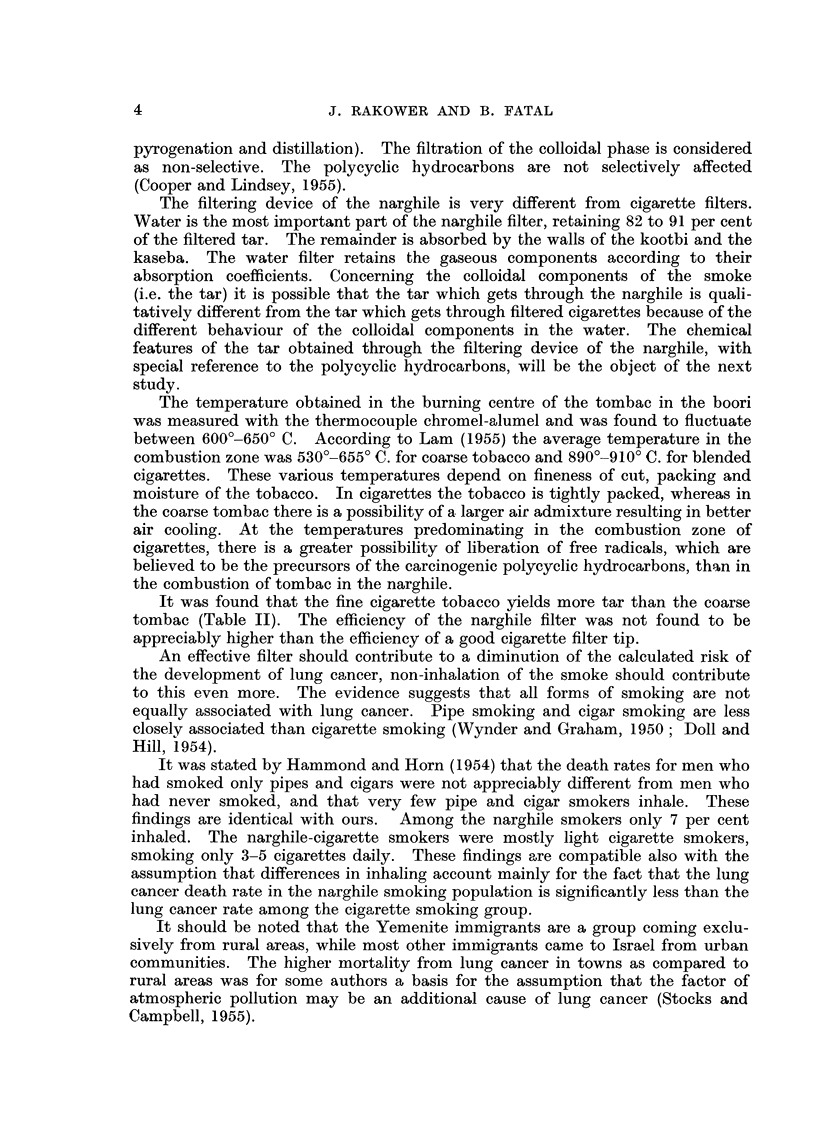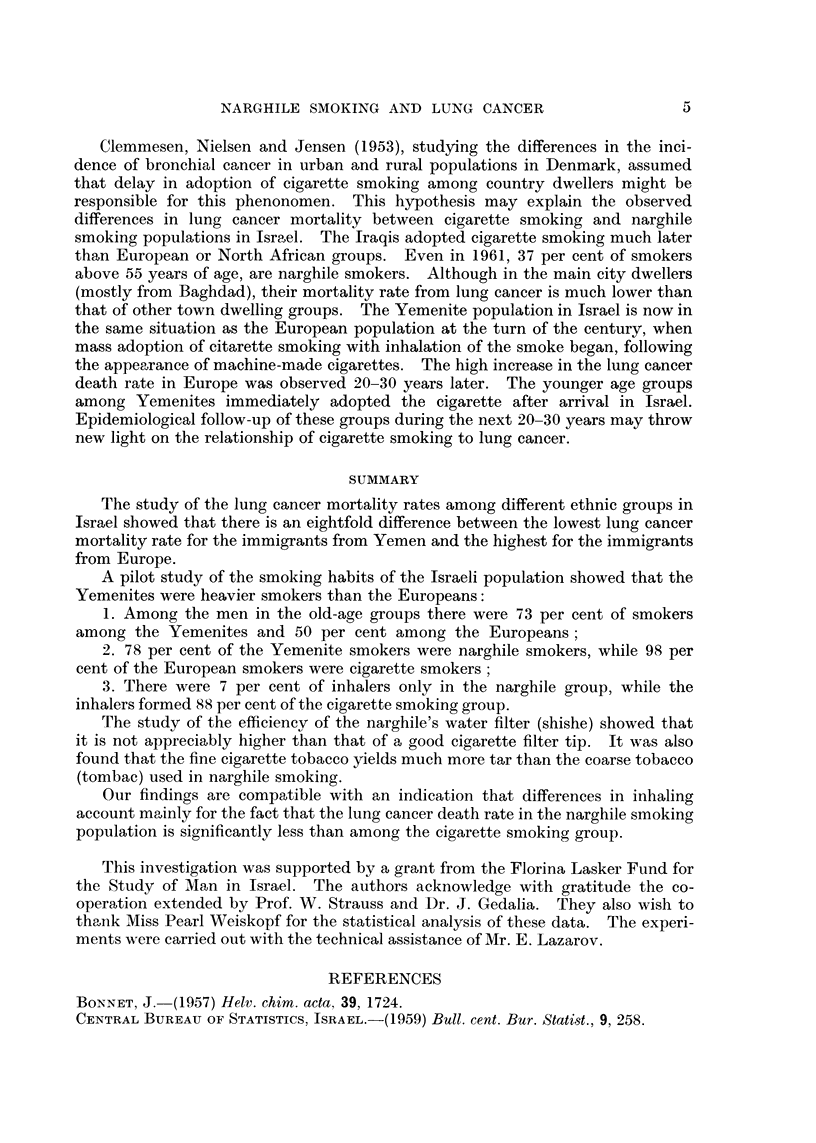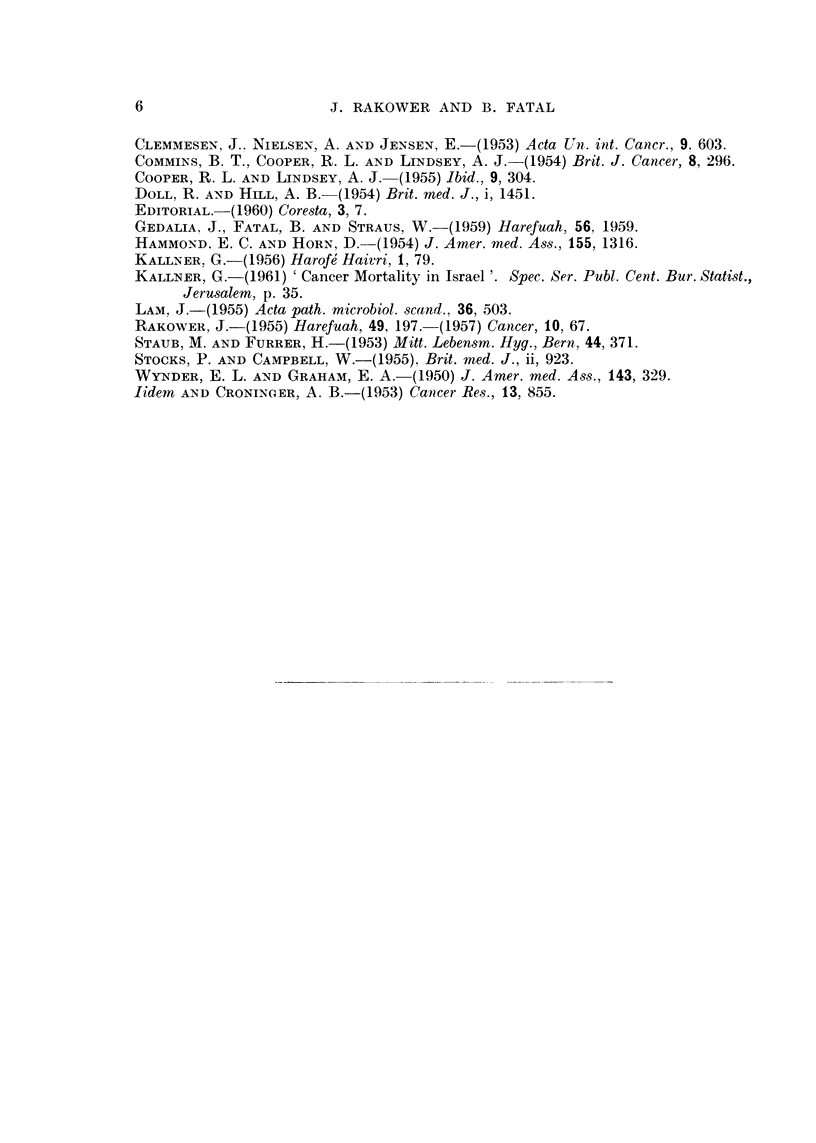# Study of Narghile Smoking in Relation to Cancer of the Lung

**DOI:** 10.1038/bjc.1962.1

**Published:** 1962-03

**Authors:** J. Rakower, B. Fatal

## Abstract

**Images:**


// BRITISH JOURNAL OF CANCER

VOL. XVI MARCH, 1962 NO. 1

### STUDY OF NARGHILE SMOKING IN RELATION TO CANCER OF THE LUNG

J. RAKOWER AND B. FATAL

*From the Department of Chest Diseases, Rothschild Hadassah University Hospital, Jerusalem, Israel, and the Department of Preventive Medicine, Hebrew University-Hadassah School of Medicine, Jerusalem, Israel*

Received for publication December 1, 1961

EPIDEMIOLOGICAL studies on mortality from lung cancer among different ethnic groups in Israel (Rakower, 1955, 1957 ; Kallner, 1956, 1961) showed a much lower mortality rate among Asiatic and African-born immigrants than among those born in Europe. The age-specific mortality rate from lung cancer among European born male immigrants was 57·3 per 100,000 as compared with 27·6 among Asiatic and African born. Further studies have shown that if this Asiatic-African born rate was broken down into individual communities, it appeared that the death rates for immigrants from Turkey (54·3) and North Africa (56·2) were on the same level as those for European Jews, while the low Oriental rate was the result of the low figures for immigrants from Iraq (24·3) and the very low value for Yemenite Jews (7·2 for 100,000) (Kallner, 1961).

Since the epidemiological studies in many countries have shown that the risk of dying from lung cancer increases in direct proportion to the consumption of cigarettes, the smoking habits of different ethnic groups of the Israeli population were investigated (Central Bureau of Statistics, 1959). These pilot studies showed that contrary to expectations, the Yemenites and Iraqis were heavier smokers than the Europeans. Among males above 55 years of age in the European group, there were 50 per cent of smokers in comparison with 60 per cent in the Iraqi and 73 per cent in the Yemenite related groups. It was shown in another study group (cf. below) that in these older age groups the Yemenite and Iraqi Jews were mostly narghile smokers, while the immigrants from Europe, Turkey and North Africa were exclusively cigarette smokers.

A study of the smoking habits of a group of 254 Yemenite males above 55 years of age showed that in Yemen 78 per cent of the smokers were exclusively narghile smokers, 16 per cent were narghile and cigarette smokers, and 6 per cent were cigarette smokers only. Among the narghile smokers only 7 per cent inhaled, while among the Europeans 88 per cent were inhalers.

With the narghile the smoke is drawn through water before reaching the mouth. The narghile consists of a boori, a kind of bowl made of clay (Yemenite narghile) or metal (Middle East narghile) (Fig. 1). In the boori the smoker puts tobacco and on the tobacco glowing charcoal. The smoke passes from the boori through

4 small holes into a pipe (kootbi) and thence into a flask of water (shishe). From the shishe the smoke reaches the smoker via a long leather tube (kaseba) measuring 3 to 4 metres in the Yemenite narghile, and 0·75 to 1 metre in the Middle East narghile.

The "tombac", i.e., the coarse tobacco used by narghile smokers, belongs to the genus *Nicotiana rustica*, known in Russia as "makhorka". The leaf is very broad, very thick and heavy and the petiole is used in smoking as well as the leaf.

The quantity used for one narghile is one fistful, i.e., 10 g. A light narghile smoker smokes 2 narghiles daily, a moderate smoker 3–4, a heavy smoker 5–7, and a chain smoker 8–10 per day.

Fig. 2.—Apparatus employed.

A. Narghile.

$B_5$–$B_1$. Washer flasks.

C. Manometer.

D. Gasometer.

E.G. Stopcocks.

H. Suction apparatus.

The purposes of this investigation is to study the efficiency of the filtering device of the narghile and particularly its water filter. As standard, the Yemenite narghile was adopted (Fig. 1*a*): the kootbi measuring 67 cm., the shishe containing 400 c.c. of water and the kaseba measuring 320 cm. in length. The narghile was fitted into a smoking apparatus (Fig. 2) in which normal narghile smoking conditions were simulated as closely as possible. Table I shows the narghile smoking parameters as compared with those of cigarette smoking as studied in different laboratories.

The smoke was absorbed in 5 flask-washers containing chloroform and sulphuric acid ($B_3$–$B_5$) or sulphuric acid only ($B_2$–$B_1$). According to an average determined by examination of the habits of many narghile smokers, a negative pressure of 30 mm. of mercury (measured by manometer C) was adopted. The

---

EXPLANATION OF PLATE.

Fig. 1.—A. Yemenite narghile. B. Middle Eastern narghile.

1. Boori—the bowl.
2. Kootbi—the stem.
3. Shishe—the vessel with water.
4. Kaseba—the tube.

1B
1
2
3
4
1A
1
2
3
4

TABLE I.—*Cigarette and Narghile Parameters and Tar Absorption*

| Author | Type of smoking | Duration of puff (seconds) | Volume of puff (c.c.) | Interval between the puffs (seconds) | Amounts of tar from 10 g. of tobacco (mg.) |
|---|---|---|---|---|---|
| Present study | Narghile (tombac) | 5 | 200 | 60 | 84 |
| Gedalia, Fatal and Strauss (1959) | Cigarettes | 2 | 40 | 30 | 260 |
| Bonnet (1957) | ,, | 2–3 | — | 30 | 390 |
| Commins, Cooper and Lindsey (1954) | ,, | 2 | 16 | 45 | 400 |
| Wynder, Graham and Croninger (1953) | ,, | 2 | 60 | 18 | 430 |
| Staub and Furrer | ,, | 2 | 40 | 15 | 720 |

vacuum was created by electrically driven suction apparatus. The volume of the puff under these conditions was 200 c.c. (measured in gasometer D). The crude weight of the collected tars was measured by the gravimetric method described by Staub and Furrer (1953). The experiment was repeated several times, 182 working samples being obtained, the accepted statistical techniques of sampling being adopted.

The amounts of tar obtained from the combustion of 10 g. of tombac and of a popular brand of tobacco through the narghile are shown in Table II. By smoking the tobacco in the boori only, it was possible to evaluate the efficiency of the narghile's filter. The following formula was adopted for the index of filter efficiency: $E = \frac{a-b}{a}$ where " a " is the total of absorbed tar from the cigarette without filter and " b " total amount of absorbed tar from the cigarette with filter (Editorial, Coresta 1960). The average efficiency index for filtering devices of the narghile was found to be 0·47. The index of efficiency found in this laboratory in different cigarette tip filters (Gedalia, Fatal and Strauss, 1959) was 0·4 for filtertips made of lignin wrapped in cotton, 0·3 for those made of crepe paper and 0·05 for filtertips made of small pieces of plain paper.

TABLE II.—*Average Quantities of Tar Obtained from Narghile Smoking of " Tombac " and Blended Cigarettes "*

| | Tar obtained from normal narghile smoking (mg.) | Tar obtained from smoking with boori only (mg.) | Tar absorbed by the narghile (mg.) | Index of narghile's filter efficiency (mg.) | Tar in the water (shishe) (mg.) | Percentage of filtered tar absorbed by water |
|---|---|---|---|---|---|---|
| Tombac 10 g. | 84 | 161 | 77 | 0·48 | 63 | 82 |
| Blended tobacco 10 g. | 142 | 262 | 120 | 0·46 | 109 | 91 |

### DISCUSSION

Tobacco smoke consists of liquid and solid particles (the disperse or the colloidal phase) which are suspended in a mixture of gases (the continuous or gaseous phase). Cigarette filters, consisting of enmeshed fibres, are assumed to be inert in relation to the gaseous components of the smoke ($N, O_2, CO, CO_2$, fumes from pyrogenation and distillation). The filtration of the colloidal phase is considered as non-selective. The polycyclic hydrocarbons are not selectively affected (Cooper and Lindsey, 1955).

The filtering device of the narghile is very different from cigarette filters. Water is the most important part of the narghile filter, retaining 82 to 91 per cent of the filtered tar. The remainder is absorbed by the walls of the kootbi and the kaseba. The water filter retains the gaseous components according to their absorption coefficients. Concerning the colloidal components of the smoke (i.e. the tar) it is possible that the tar which gets through the narghile is qualitatively different from the tar which gets through filtered cigarettes because of the different behaviour of the colloidal components in the water. The chemical features of the tar obtained through the filtering device of the narghile, with special reference to the polycyclic hydrocarbons, will be the object of the next study.

The temperature obtained in the burning centre of the tombac in the boori was measured with the thermocouple chromel-alumel and was found to fluctuate between 600°–650° C. According to Lam (1955) the average temperature in the combustion zone was 530°–655° C. for coarse tobacco and 890°–910° C. for blended cigarettes. These various temperatures depend on fineness of cut, packing and moisture of the tobacco. In cigarettes the tobacco is tightly packed, whereas in the coarse tombac there is a possibility of a larger air admixture resulting in better air cooling. At the temperatures predominating in the combustion zone of cigarettes, there is a greater possibility of liberation of free radicals, which are believed to be the precursors of the carcinogenic polycyclic hydrocarbons, than in the combustion of tombac in the narghile.

It was found that the fine cigarette tobacco yields more tar than the coarse tombac (Table II). The efficiency of the narghile filter was not found to be appreciably higher than the efficiency of a good cigarette filter tip.

An effective filter should contribute to a diminution of the calculated risk of the development of lung cancer, non-inhalation of the smoke should contribute to this even more. The evidence suggests that all forms of smoking are not equally associated with lung cancer. Pipe smoking and cigar smoking are less closely associated than cigarette smoking (Wynder and Graham, 1950 ; Doll and Hill, 1954).

It was stated by Hammond and Horn (1954) that the death rates for men who had smoked only pipes and cigars were not appreciably different from men who had never smoked, and that very few pipe and cigar smokers inhale. These findings are identical with ours. Among the narghile smokers only 7 per cent inhaled. The narghile-cigarette smokers were mostly light cigarette smokers, smoking only 3–5 cigarettes daily. These findings are compatible also with the assumption that differences in inhaling account mainly for the fact that the lung cancer death rate in the narghile smoking population is significantly less than the lung cancer rate among the cigarette smoking group.

It should be noted that the Yemenite immigrants are a group coming exclusively from rural areas, while most other immigrants came to Israel from urban communities. The higher mortality from lung cancer in towns as compared to rural areas was for some authors a basis for the assumption that the factor of atmospheric pollution may be an additional cause of lung cancer (Stocks and Campbell, 1955).

Clemmesen, Nielsen and Jensen (1953), studying the differences in the incidence of bronchial cancer in urban and rural populations in Denmark, assumed that delay in adoption of cigarette smoking among country dwellers might be responsible for this phenonomen. This hypothesis may explain the observed differences in lung cancer mortality between cigarette smoking and narghile smoking populations in Israel. The Iraqis adopted cigarette smoking much later than European or North African groups. Even in 1961, 37 per cent of smokers above 55 years of age, are narghile smokers. Although in the main city dwellers (mostly from Baghdad), their mortality rate from lung cancer is much lower than that of other town dwelling groups. The Yemenite population in Israel is now in the same situation as the European population at the turn of the century, when mass adoption of citarette smoking with inhalation of the smoke began, following the appearance of machine-made cigarettes. The high increase in the lung cancer death rate in Europe was observed 20–30 years later. The younger age groups among Yemenites immediately adopted the cigarette after arrival in Israel. Epidemiological follow-up of these groups during the next 20–30 years may throw new light on the relationship of cigarette smoking to lung cancer.

SUMMARY

The study of the lung cancer mortality rates among different ethnic groups in Israel showed that there is an eightfold difference between the lowest lung cancer mortality rate for the immigrants from Yemen and the highest for the immigrants from Europe.

A pilot study of the smoking habits of the Israeli population showed that the Yemenites were heavier smokers than the Europeans:

1. Among the men in the old-age groups there were 73 per cent of smokers among the Yemenites and 50 per cent among the Europeans ;

2. 78 per cent of the Yemenite smokers were narghile smokers, while 98 per cent of the European smokers were cigarette smokers ;

3. There were 7 per cent of inhalers only in the narghile group, while the inhalers formed 88 per cent of the cigarette smoking group.

The study of the efficiency of the narghile's water filter (shishe) showed that it is not appreciably higher than that of a good cigarette filter tip. It was also found that the fine cigarette tobacco yields much more tar than the coarse tobacco (tombac) used in narghile smoking.

Our findings are compatible with an indication that differences in inhaling account mainly for the fact that the lung cancer death rate in the narghile smoking population is significantly less than among the cigarette smoking group.

This investigation was supported by a grant from the Florina Lasker Fund for the Study of Man in Israel. The authors acknowledge with gratitude the cooperation extended by Prof. W. Strauss and Dr. J. Gedalia. They also wish to thank Miss Pearl Weiskopf for the statistical analysis of these data. The experiments were carried out with the technical assistance of Mr. E. Lazarov.

REFERENCES

BONNET, J.—(1957) *Helv. chim. acta*, **39**, 1724.

CENTRAL BUREAU OF STATISTICS, ISRAEL.—(1959) *Bull. cent. Bur. Statist.*, **9**, 258.

CLEMMESEN, J., NIELSEN, A. AND JENSEN, E.—(1953) *Acta Un. int. Cancr.*, **9**, 603.
COMMINS, B. T., COOPER, R. L. AND LINDSEY, A. J.—(1954) *Brit. J. Cancer*, **8**, 296.
COOPER, R. L. AND LINDSEY, A. J.—(1955) *Ibid.*, **9**, 304.
DOLL, R. AND HILL, A. B.—(1954) *Brit. med. J.*, i, 1451.
EDITORIAL.—(1960) *Coresta*, **3**, 7.
GEDALIA, J., FATAL, B. AND STRAUS, W.—(1959) *Harefuah*, **56**, 1959.
HAMMOND, E. C. AND HORN, D.—(1954) *J. Amer. med. Ass.*, **155**, 1316.
KALLNER, G.—(1956) *Harofé Haivri*, **1**, 79.
KALLNER, G.—(1961) ' Cancer Mortality in Israel '. *Spec. Ser. Publ. Cent. Bur. Statist., Jerusalem*, p. 35.
LAM, J.—(1955) *Acta path. microbiol. scand.*, **36**, 503.
RAKOWER, J.—(1955) *Harefuah*, **49**, 197.—(1957) *Cancer*, **10**, 67.
STAUB, M. AND FURRER, H.—(1953) *Mitt. Lebensm. Hyg., Bern*, **44**, 371.
STOCKS, P. AND CAMPBELL, W.—(1955), *Brit. med. J.*, ii, 923.
WYNDER, E. L. AND GRAHAM, E. A.—(1950) *J. Amer. med. Ass.*, **143**, 329.
*Iidem* AND CRONINGER, A. B.—(1953) *Cancer Res.*, **13**, 855.